# A comprehensive evaluation of the potential of semiterrestrial isopods, *Ligia exotica*, as a new animal food

**DOI:** 10.1038/s41598-021-86561-z

**Published:** 2021-03-30

**Authors:** Lele Xu, Yongqin Li, Yao Liu, Haifeng Mi, Xiang Jiang, Yulin Sun, Haiyong Zhao, Daohai Chen, Liyun Wang

**Affiliations:** 1 Engineering Technology Research Center for Sustainable Utilization of Seafood Resources in Western Guangdong Province, Zhanjiang, China; 2 Life Science and Technology School, Lingnan Normal University, Zhanjiang, China; 3 School of Information Engineering, Lingnan Normal University, Zhanjiang, China; 4 Fisheries Institute, Tongwei Group Co., Ltd., Chengdu, China; 5 School of Foreign Studies, Capital University of Economics and Business, Beijing, China

**Keywords:** Zoology, Animal physiology

## Abstract

The semiterrestrial isopod, *Ligia exotica* represents one of the oldest documented species introductions of marine organisms and is known as an intermediate form between marine and strictly terrestrial isopods. In order to explore the potential value of *Ligia* as an animal food source, this study focused on the growth rate under laboratory rearing conditions and conducted a detailed analysis of the overall nutrient content of the species in comparison to two other marine food media (krill and fish meal). Evaluation of the growth rate of juveniles suggests it is a relatively fast-growing species of the *Ligiidae* family. The essential amino acids content *Ligia* meal is the lowest amongst the three studied media but the proportion of flavor amino acids, and in particular taurine, was higher. The most restricted amino acids of isopod meal are methionine and cysteine. The significantly unbalanced amino acid composition of *Ligia* meal may affect the absorption and utilization by consumers. In terms of fatty acids, the total polyunsaturated fatty acids in the isopod is very low. A total of 12 vitamins were examined. The VK_1,_ VE, VB_2_, VB_3_, VB_5_ content of isopod meal were significantly higher than those of krill meal and fish meal. Similarly, most of the 11 mineral elements are highest in the isopod meal. *Ligia* therefore offers potential as an alternative natural food source in animal given the growth rate under culture and the overall nutrient content. But *Ligia* collected in most of the field would be deemed unfit for human consumption because of the relatively low nutritional value and heavy metal content exceeding the provided standard. Further study is warranted to elucidate the biological characteristics of isopods and how its diet is reflected in its nutritional value to consumers.

## Introduction

**Figure 1:**
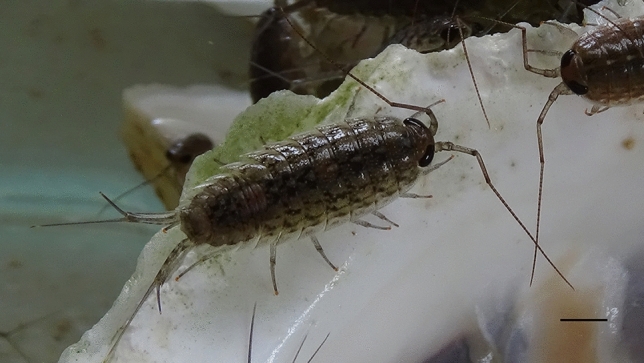
External morphology of *Ligia exotica* (scale bar = 0.6 cm).

*Ligia* is a genus of isopods (Isopoda; Crustacea), which is commonly known as rock lice or sea slaters based on its appearance (Fig. 1). Most *Ligia* species live on tidal zone cliffs and rocky beaches, as well as dams, ports and docks and tolerate a wide range of temperatures and salinity. They are distributed across almost the entire coastline of East Asia^1^ and have naturally high biomass. Coastal *Ligia* exhibits a mixture of terrestrial and marine characteristics, drying out easily, needing moist air and proximity to water. Although they have gills and can respire under water, they only submerge when escaping terrestrial predators or being dislodged by wave action. They are well adapted to rocky surfaces but avoid sand, which exposes them to terrestrial predation and desiccation^2^. The fertilized eggs of *L. exotica* develop into juveniles in the brood pouch (built from oöstegites) of females until they can live independently. It takes about 5 weeks from egg deposition to release^3^. *Ligia* isopods show many important adaptations for their lifestyle. Individuals can osmo-regulate well and are found in full salt water habitats to near fresh water seeps area^4^. They store calcium in CaCO_3_ deposits visible as white regions in the anterior sternites to fulfill the biphasic moult, which is different with most crustaceans^5^. Besides, *Ligia* transports water only by using open capillaries in its legs containing hair- and paddle-like microstructures, and this passive water transport mechanism may inspire novel biomimetic fluid manipulations with or without a gravitational field^6^, etc. More details can be found in an academic website A Snail’s Odyssey^7^, and in other articles^8–10^.

Traditionally, the taxonomy of *Ligia* isopods has mainly been based on morphological characteristics but recently the study of intraspecific genetic differentiation using molecular systematics has provided a new understanding of the systematic classification of *Ligia* species. Santamaria et al.^11^ revealed a surprisingly constrained body shape among highly genetically divergent allopatric lineages of *Ligia occidentalis* and cryptic lineages within *Ligia* species in the region of Seychelles islands^12^, southern Africa coastline^13^, Hawaiian Islands^14^ have been uncovered. Santamaria et al.^15^ also recorded the *Ligia baudiniana* species complex in the American Gulf of Mexico coastline by morphological and molecular approaches. According to these molecular taxonomic studies, three research hotspots were suggested by Huang et al.^16^, including Pan-California Peninsula group, Hawaii Islands group and China-Japan-Korea East Asia group. Japanese islands have certain features in common with the Seychelles and Hawaii islands, where geographical isolation has led to the high species diversity of Ligiidae. Within this family *Ligia exotica* is the most widely distributed species.

*Ligia* isopods are omnivorous detrivores and fed by chewing on organic debris on the shore, who preferred wrack over fresh debris^17^. *Ligia* isopods themselves are often used as bait by fishermen and they are the primary prey for mangrove crabs (personal field observation), marine fish, birds *Copsychus saularis*^18^, and lizards^19^ and even small mammals^20^. *Ligia* are considered to play an active role in nutrient recycling and energy flow in the near shore environment and in supporting the biodiversity of the coastal zone^16^.

Our preliminarily study has confirmed that *Ligia exotica* can provide a high-quality natural diet for cultured cuttlefish *Sepia pharaonis*^21^. Since *Ligia exotica* is both a grazer and a scavenger on plant remains and detritus, and is easily adaptive to the environment, the cost of being cultured should far less than that of live shrimp, which seriously affects the profitability of cuttlefish farming. However, it is still unknown how long this sole diet could support the growth of juvenile cuttlefish, when the feeding trial just lasts for days. On the other hand, *Ligia* isopods are also utilized in traditional Chinese medicine for the treatment of muscle injury, swelling and pain, or to overcome malnutrition in children^22^. Extracts from *Ligia exotica* were proven to have obvious proliferation inhibitory effects on a range of biochemical and cellular functions such as cervical cancer cells HeLa, stomach cancer cell SGC-7901 and NCI-60 human tumor cell in vitro and have an inhibitory effect on mouse sarcoma S180-induced transplantable cancers by intraperitoneal injection in tumors over 7d^23^. A novel nucleoside, elucidated as 3′-*O*- (α-D-glucosyl) inosine, had been isolated from *Ligia exotica* but its bioactivity was not identified^24^. Similarly, two novel aspochalasins have been isolated from the gut fungus which was found in marine isopod *Ligia oceanica*^25^. Yue et al.^26^ not only demonstrated the pharmaceutical value of *L. exotica* for pain-relief in Chinese folk medicine, but also suggested that sea slaters may represent a promising source for discovery of novel analgesic and anti-inflammatory compounds in near future.

As far as we are aware, there is no published nutritional analysis of *Ligia* isopods although there is evidence of potential nutritional for animal as well as medicinal value. Hence we established a 70-day culture experiment on juveniles of *Ligia* to evaluate their growth performance and conducted a detailed analysis of the nutritional content of *Ligia exotica* meal compared with two others regularly used marine food sources, white fish meal and Antarctic krill (*Euphausia superba*) meal. The objectives included whether *Ligia* can be artificially cultured on a large scale and to document their growth rate and how well the nutritional value is to animals compared with the reference food sources.

## Materials and methods

### Growth rate determination of juveniles *Ligia exotica*

*Ligia exotica* specimen were collected at the embankment of Tiaoshun Island in Zhanjiang City, Guangdong Province, China (N 21° 28′, E 110° 39′) in June to September 2017. The species identification was confirmed by the morphological character, which usually has 37–40 segmental number in 2nd antenna^27^. Dozens of adults were cultured in a 40 cm × 20 cm × 30 cm aquarium, with oyster shells stacked on the right side and a plastic baffle with small holes through which seawater could pass was fixed to the bottom left 10 cm of the aquarium. Sea water reached half the height of the oyster stack and a filter pump was installed. *Ligia exotica* were fed daily with tilapia fish pellet feed placed on dry oyster shells.

When gravid females were observed, especially where fertilized eggs in the marsupium were found to change color from orange to black, they were immediately isolated into a plastic box with a layer of cotton covered with a layer of gauze on the bottom. A piece of paper was placed on the gauze and thoroughly wetted with clean seawater. Excessive tilapia pellet feed was spread on the paper as a food source. The cotton, gauze and seawater were changed every two days.

When sufficiently developed, the 50–60 juveniles crawled out from the marsupium of the brooding female. The time of birth was recorded, and the mothers removed from the plastic box to avoid cannibalism. Juveniles from each female were divided into groups of ten individuals and cultured in a plastic test tube separately and in a constant temperature incubator at 28 °C. Hence the 50–60 juveniles were divided evenly into 5–6 groups, which consist of 5–6 check points waited to be sampled. A total of 15 check points for 70 days lasted culture experiment were established in this way and cultured under similar conditions as described that of gravid females.

Each group of juveniles was weighted every 5 days. After being frozen at − 20 °C, the samples were placed at room temperature for 20 min to volatilize the water on the body surface and were weighed together with a high-precision electronic balance. The total weight of ten juveniles then transformed into the value of average individual weight that facilitate to growth analysis. The check points were set up in triplicate and the sampling procedures was performed as described above.

### Analysis of nutritional components of *Ligia exotica* and comparative substrates

The frozen field collected *Ligia exotica* were subsequently dried at 75 °C for one day in an oven, ground into powder and stored at − 20 °C until analysis.

Two readily available aquatic food substrates were used for comparative purposes. Antarctic krill (*Euphausia superba*) meal was purchased from the China National Fisheries Corporation. It had originally been cooked at 80 ~ 95 °C for 20 ~ 25 min, dehydrated and dried on board when caught at sea and was stored in the laboratory at − 20 °C.

Fishmeal was white fish meal (degreased) imported from Russia, PJSC Nakhodka Active Marine Fishery Base, which was processed directly on board and mainly composed of the pacific cod *Gadus macrocephalus*. When delivered to the laboratory, the samples were divided into several bags, stored at − 20 °C and sampled at random during the experiment.

A range of nutrient components were analyzed from *Ligia* and the comparative substrates as described in Supplementary Table S1 online.

### Evaluation of nutritional quality of amino acids

Based on the amino acid scoring standard model recommended by FAO & WHO^28^ and the amino acid model using egg protein as an ideal protein reference, the Amino Acid Score (AAS), Chemical Score (CS) and Essential Amino Acid Index (EAAI) from eight essential amino acids for humans were calculated from the following formulae^29^. The higher the scores and indices that the substrates received, the more similarity they are with the ideal protein model, and the better the protein quality for human consumption.

1 $$AAS=\frac{aa}{AA\;\mathrm{FAO}\;\&\;\mathrm{ WHO}}$$

2 $$CS=\frac{aa}{AA\;\mathrm{Egg}}$$

3 $$\mathrm{EAAI}=\sqrt[n]{\frac{100A}{AE}\times \frac{100B}{BE}\times \frac{100C}{CE}\times \dots \times \frac{100I}{IE}}$$

where *aa* is the amino acid content of the sample (%); *AA*_FAO&WHO_ is the content of the same amino acid recommended by FAO & WHO (%) (see Table 3); *AA*_egg_ is the content of the same amino acid in whole egg protein (%); n is the number of essential amino acids compared (n = 9). A, B, C, ⋯; I is the content of essential amino acid of sample protein (mg/g N), AE, BE, CE, ⋯; IE is the content of essential amino acid of whole egg protein (mg/g N).

### Statistical analysis

As far as the juveniles development/growth analysis is concerned, by using the function of data regression analysis in Microsoft Excel software, a power function regression model with individual body weight on ages was established, in order to obtain the growth curve (trendline) and the regression determination coefficient (*r*^2^). Meanwhile the residual statistics was conducted in SPSS 26.0 to test whether the weight gain values follow the normal distribution.

Following the Chinese national determination standard method, the analysis of each samples was repeated three times by the same tester to obtain data for statistical analysis. When conducting the fatty acid and vitamin content analysis, the concentration of some parameters that were too low to be detected (ND) and were considered zero with no statistical analysis undertaken. The normality of the original raw data was confirmed by the Shapiro–Wilk method in SPSS 26.0 prior to statistical analysis. It indicated that all the original data follow the normal distribution.

Levene’s test was adopted to test the homogeneity of data of the nutritional parameters of the three food materials (isopod meal, antarctic krill meal and fishmeal), in advance for Analysis of Variance (ANOVA, two tailed). In the case of homogeneity of variances, Duncan’s multiple range test (multiple F test) was used to identify any difference in mean values. Meanwhile Fisher’s least significant difference (LSD) was employed (assuming that isopod meal is controlled and then compare it respectively with krill and fish meal) as references to verify the statistical differences. If the data violated the assumption of homogeneity of variances, Welch's Anova was used and post-hoc methods of Dunnett's T3 test employed to identify the significance or otherwise of the differences. *P* < 0.05 was considered significant. Mean ± 95% confidence interval (constructed with *t*-distribution) was used to describe the statistical data.

**Figure 2:**
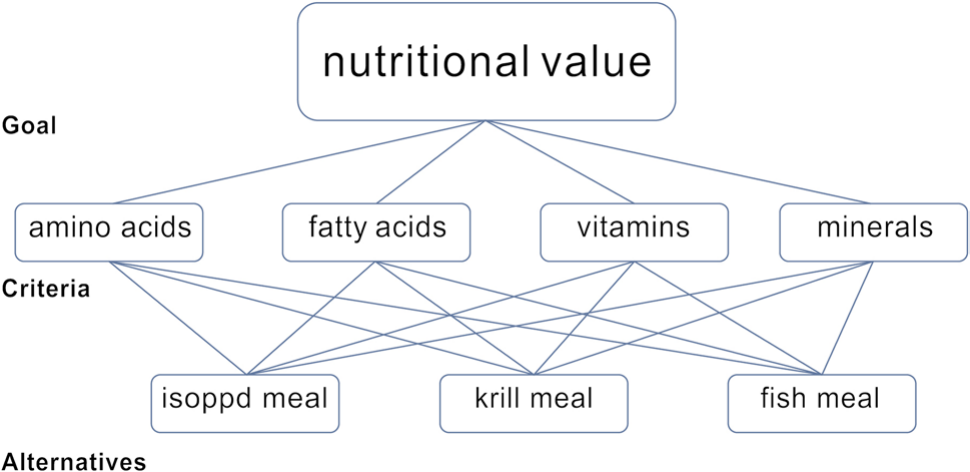
Analytical hierarchy process model of nutritional value for *L.exotica*, krill meal and fish meal.

By employing the analytic hierarchy process (AHP) technique, a structural analysis model was established for evaluating the nutritional value of fish meal, isopod meal and krill powder in relation to amino acids, fatty acids, vitamins and minerals (Fig. 2).

Nutritional value was assessed based on the considered views of 3 nutritional experts in a small advisory committee, including expertise in human nutrition education (Lingnan Normal University. China), in swine nutrition (Jiangsu AnYou Biotechnology Group Co., Ltd. China), and in aquaculture nutrition (Ocean University of China, China). According to the scoring criteria in Table 1, each expert rated the nutritional components of the substrates and assessed the accuracy of the nutritional value judged by the four nutritional indicators, i.e., amino acids, fatty acids, vitamins and minerals.

**Table 1:** Table of relative scores.

Value of *j* & k*	Interpretation
1	*j* and *k* are equally important
3	*j* is slightly more important than *k*
5	*j* is more important than *k*
7	*j* is strongly more important than *k*
9	*j* is absolutely more important than *k*
2, 4, 6, 8	intermediate value in two adjacent judgments

*j & *k* not only mean the comparison between different nutritional indicators, but also the comparison between same indicators in different substrates.

The judgment matrices of each expert were imported into the group decision system and tested for consistency by YAAHP (Yet Another AHP) V.10.0 software. Upon testing, all the matrices derived from the scores of three experts met the consistency requirement (consistency ratio = 0.0981, 0.0000, 0.0398, respectively). The total sequencing weight value was calculated through arithmetical average, which was generated from the matrices provided by experts.

### Animal welfare statement

The authors confirm that they have followed EU standards for the protection of animals used for scientific purposes.

### Ethics approval and consent to participate

The study received approval from the institutional review board of Lingnan Normal University.

### Consent for publication

The authors give our permission for the following manuscript to appear in the print, online, and licensed versions of *Scientific Reports* and for the Journal to grant permission to third parties to reproduce this manuscript.

## Results

### Growth performance of juvenile *Ligia exotica*

**Figure 3:**
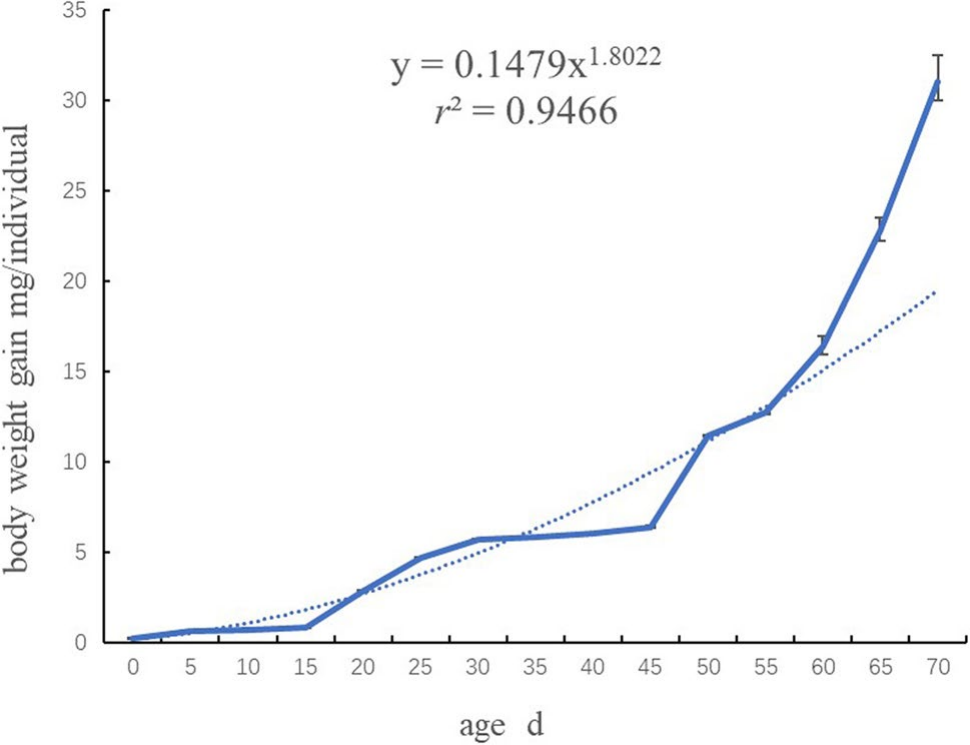
Growth curve (body weight gain over time) of juvenile *Ligia exotica.* n = 3 groups, each contain ten individuals from the same female. Dotted line is the trendline that generated in Microsoft Excel (https://docs.microsoft.com/zh-cn/microsoft-365/?view=o365-worldwide). Microsoft 365 Apps for enterprise, Microsoft, USA. ± Bar means 95% confidence interval.

The average body weight of the new-hatched juveniles of *Ligia exotica* is 0.24 ± 0.005 mg. The increase of body weight (BW) in the early stages is not significant until 15 days after hatching (DAH), and after which BW increases from 0.85 ± 0.02 mg to 6.37 ± 0.04 mg at 45 DAH (Fig. 3). From then on, the weight gain of individuals begins to accelerate significantly, reaching 12.69 ± 0.01 mg at 55 DAH and 16.37 ± 0.41 mg at 60 DAH. This data fits the equation of power function y = 0.1479×1.8022, while *r*^2^ = 0.9466. Standardized residual plots showed that almost of the scatter points fall on the diagonal of the normal P-P plot, meaning the weight gain values follow the normal distribution. The final BW value of *Ligia* at 70 DAH is 31.06 ± 1.06 mg. Weight gain rate of juvenile *Ligia* is ((final BW- initial BW)/initial BW × 100%) is 13026.76%, and specific growth rate per day is ((ln final BW—ln initial BW )/DAH × 100%) is 6.97%.

### General nutritional components of *Ligia*

**Figure 4:**
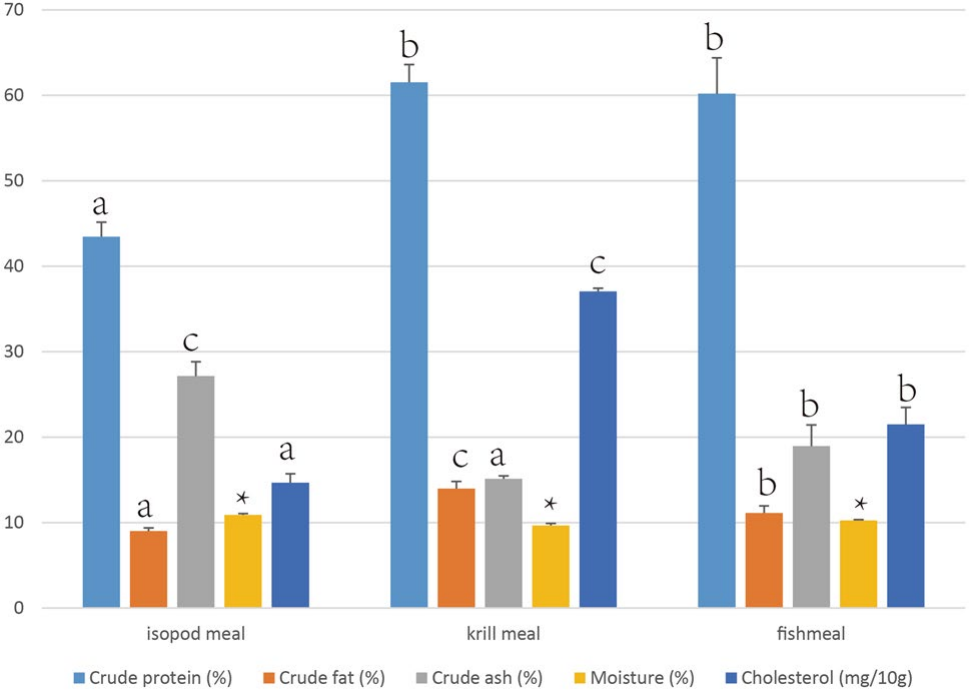
General nutritional components of isopod meal, antarctic krill meal and fishmeal (wet weight ± 95% confidence interval). Numerical values marked in the top of column with the same letter ^a, b^ or ^c^ or * are not statistically significantly different.

As shown in Fig. 4, the crude protein of isopod meal as a percentage of wet weight is less than both the krill meal and fishmeal (F = 225.18, df = 2). The crude fat (F = 224.02) and cholesterol content (F = 1430.29) of the isopod meal is also lower whereas the crude ash content (F = 237.64) is higher than both krill and fishmeal.

### Amino acids composition

Table 2 shows the composition and content of 18 amino acids and taurine in isopod meal, antarctic krill meal and fish meal. The total amino acid content (ΣAA) of isopod meal is higher than that of krill meal but significantly lower than that of fish meal (F = 989.81, all df = 2). The contents of the nine essential amino acids (ΣEAA) differ with the lowest values found in *Ligia* (F = 181.36). Also, the ratio of essential to total amount of amino acids (ΣEAA/ΣAA) of isopod meal is the lowest of the three substrates (F = 17.92). Surprisingly however, the content of taurine, a beneficial non-protein amino acid, is much greater in *Ligia* than that of krill meal and fish meal (F = 784.36). The content of five flavored amino acids is higher in the isopod meal as well than that of krill powder (F = 91.28), and its proportion to the total amino acid (ΣFAA/ΣAA) is also higher than both of krill meal and fish meal (F = 51.10).

**Table 2:** Amino acids composition of isopod meal, antarctic krill meal and fish meal (%, dry weight).

Amino acids (g/100 g)	Isopod meal	Krill meal	Fish meal
Aspartate (Asp)^f^	4.16 ± 0.07^a^	4.97 ± 0.10^b^	6.68 ± 0.05^c^
Threonine (Thr)^e^	1.86 ± 0.05^a^	1.92 ± 0.07^b^	2.98 ± 0.07^c^
Serine (Ser)	1.38 ± 0.12^a^	2.02 ± 0.05^b^	3.18 ± 0.05^c^
Glutamate (Glu) ^f^	4.66 ± 0.05^a^	7.06 ± 0.05^b^	9.62 ± 0.01^c^
Glycine (Gly)^f^	6.96 ± 0.05^c^	2.04 ± 0.05^a^	4.43 ± 0.07^b^
Alanine (Ala)^f^	2.11 ± 0.07^a^	2.56 ± 0.12^b^	4.35 ± 0.07^c^
Valine (Val)^e^	3.90 ± 0.10^c^	2.38 ± 0.07^a^	3.44 ± 0.05^b^
Methionine (Met)^e^	0.54 ± 0.02^a^	1.46 ± 0.05^b^	1.72 ± 0.10^c^
Isoleucine (Ile)^e^	1.19 ± 0.05^a^	2.41 ± 0.05^b^	2.91 ± 0.10^c^
Leucine (Leu)^e^	4.26 ± 0.05^b^	3.90 ± 0.02^a^	5.50 ± 0.07^c^
Tyrosine (Tyr)^e^	1.76 ± 0.02^b^	1.68 ± 0.01^a^	2.40 ± 0.02^c^
Phenylalanine (Phe)^e^	1.84 ± 0.05^a^	2.39 ± 0.10^b^	2.84 ± 0.02^c^
Lysine (Lys)^e^	3.36 ± 0.05^a^	3.72 ± 0.02^b^	5.39 ± 0.10^c^
Histidine (His)	0.99 ± 0.10^a^	2.01 ± 0.02^c^	1.62 ± 0.05^b^
Arginine (Arg)	4.24 ± 0.01^b^	2.94 ± 0.05^a^	4.50 ± 0.07^c^
Proline (Pro)	2.37 ± 0.07^b^	1.68 ± 0.10^a^	3.15 ± 0.01^c^
Cysteine (Cys)^e^	0.09 ± 0.07^a^	1.34 ± 0.05^b^	1.37 ± 0.05^b^
Tryptophan (Trp)^e^	0.61 ± 0.02^a^	0.88 ± 0.10^b^	0.62 ± 0.02^a^
taurine^f^	9.45 ± 0.20^c^	2.93 ± 0.10^b^	2.04 ± 0.12^a^
ΣAA	55.61 ± 1.91^b^	50.11 ± 0.92^a^	68.55 ± 0.75^c^
ΣEAA	19.57 ± 1.44^a^	20.73 ± 1.69^b^	27.74 ± 1.04^c^
ΣNEAA	36.73 ± 1.41^b^	29.41 ± 0.45^a^	40.63 ± 2.66^c^
ΣFAA	27.45 ± 1.17^b^	19.54 ± 2.24^a^	27.28 ± 0.27^b^
ΣEAA/ΣAA (%)	35.21 ± 1.14^a^	41.38 ± 2.51^b^	40.47 ± 1.24^b^
ΣEAA/ΣNEAA (%)	53.29 ± 2.78^a^	70.52 ± 5.14^b^	68.34 ± 2.01^b^
ΣFAA/ΣAA (%)	49.37 ± 1.19^a^	38.98 ± 4.37^b^	39.80 ± 0.45^b^

*ΣAA* is total amino acids, *ΣEAA* is total essential amino acids, *Σ NEAA* is total nonessential amino acids, and *ΣFAA* is total flavor amino acids.

Amino acids marked ^e^means essential amino acids, while ^f^means flavor amino acid.

Numerical values marked with the same letter ^a,b^ or ^c^are not statistically significantly different.

### Nutritional evaluation of amino acids

The amino acid score (AAS), chemical score (CS, the limiting amino acid index) and essential amino acid index (EAAI) were calculated by converting the data in Table 2 into milligrams of amino acid per gram of nitrogen (× 62.5). The results were compared with the amino acid scoring standard pattern suggested by FAO/WHO and the standard amino acid pattern of whole egg protein.

Amino Acid Score (AAS) and Chemical Score (CS) reflect the relationship of protein composition and utilization ratio from different perspectives. As can be seen from Table 3, in most of the case, the lowest scores are from the isopod meal, with Ile and Met + Cys in particular less than half of the value of krill meal and fishmeal. Isoleucine, Methionine and cystine are therefore the main limiting amino acids of isopod as suggested by their content. EAAI index reflects how close the essential amino acid content of material is to the standard protein (egg protein). Comparing the values of EAAI indicates that the protein quality of the isopod meal is worse than that of both krill meal and fishmeal. The high AAS and CS scores of fishmeal demonstrate that fishmeal is rich in essential amino acids and it is well-balanced in composition.

**Table 3:** Comparative analysis of amino acid score (AAS), chemical score (CS) and essential amino acid index (EAAI) of antarctic krill meal and isopod meal.

Amino acids		FAO evaluation standard (mg/gN)	Egg protein standard (mg/gN)	Score of isopod meal	Score of krill meal	Score of fish meal
AAS	Ile	250		0.30	0.60	0.73
	Leu	440		0.61	0.55	0.78
	Lys	340		0.62	0.68	0.99
	Thr	250		0.47	0.48	0.75
	Val	310		0.79	0.48	0.69
	Trp	60		0.64	0.92	0.65
	Met + Cys	220		0.18	0.80	0.88
	Phe + Tyr	380		0.59	0.67	0.86
CS	Ile		331	0.22	0.46	0.55
	Leu		534	0.50	0.46	0.64
	Lys		441	0.48	0.53	0.76
	Thr		292	0.40	0.41	0.64
	Val		410	0.59	0.36	0.53
	Trp		106	0.36	0.52	0.37
	Met + Cys		386	0.10	0.45	0.50
	Phe + Tyr		565	0.40	0.45	0.58
EAAI				34.13	45.16	55.93

### Nutritional composition of fatty acid

Table 4 shows the fatty acid composition of the three substrates. There are 12 fatty acids including 3 saturated fatty acids (SFA), 3 monounsaturated fatty acids (MUFA) and 6 polyunsaturated fatty acids (PUFA) in isopod meal. 13 fatty acids were detected in krill meal, including 4 SFA, 3 MUFA,6 PUFA, while all 17 fatty acids (4 SFA,6 MUFA and 7 PUFA) were found in fish meal. The actual content of saturated fatty acid (SFA) in isopod meal is similar to that of krill but higher than that in fish meal (F = 18.00, df = 2). The content of monounsaturated fatty acids (MUFA, F = 71.11), and EPA and DHA are the lowest in the isopod (F = 367.63 and F = 311.70 respectively) and although the content of n-6 PUFA is slightly higher in the isopod than that of krill meal (F = 55.69), the total content of PUFA is far lower than either krill meal or fishmeal (F = 117.81).

**Table 4:** Fatty acids content of isopod meal, Antarctic krill meal and fishmeal.

Fatty acids	Isopod meal	Krill meal	Fishmeal
C14:0/C16:0	1.95 ± 0.10^a^/27.33 ± 0.02^a^	8.90 ± 0.05^c^/22.89 ± 0.02^b^	5.90 ± 0.10 ^b^/17.32 ± 0.05^c^
C17:0/C18:0	None/4.39 ± 0.05^c^	0.26 ± 0.07/1.20 ± 0.05^a^	0.58 ± 0.05/2.78 ± 0.02^b^
Σ SFA	33.66 ± 4.99^b^	33.13 ± 3.00^b^	26.88 ± 3.16^a^
C16:1/C17:1	11.22 ± 0.07^c^/None	6.93 ± 0.05^a^/None	8.31 ± 0.07^b^/0.32 ± 0.05
C18:1 n9 C/C20:1 n9	7.48 ± 0.02^a^/None	18.0 ± 0.05^c^/1.64 ± 0.05^b^	15.29 ± 0.02^b^/0.85 ± 0.07^a^
C22:1 n9/C24:1 n9	None/0.57 ± 0.05	None/None	1.19 ± 0.05/0.81 ± 0.12
Σ MUFA	19.28 ± 3.03^a^	26.57 ± 1.14^b^	26.61 ± 1.86^b^
C18:2 n6 c/C18:3 n6	10.11 ± 0.10^c^/0.33 ± 0.02^c^	3.10 ± 0.12^b^/0.40 ± 0.02^b^	1.79 ± 0.05^a^/0.04 ± 0.01^a^
C18:3 n3	3.41 ± 0.05^c^	0.94 ± 0.05^b^	1.05 ± 0.02^a^
C20:2 n6/C20:3 n6	0.5 ± 0.05^a^/0.29 ± 0.05^a^	4.21 ± 0.10^c^/0.32 ± 0.07^a^	2.06 ± 0.07^b^/12.94 ± 0.10^b^
C20:3 n3/C20:4 n6	0.31 ± 0.05/None	None/0.51 ± 0.15	0.31 ± 0.02/0.70 ± 0.05
EPA C20:5 n3	6.54 ± 0.02^a^	18.30 ± 0.05^c^	14.70 ± 0.05^b^
DPA C22:5 n3	0.62 ± 0.05^b^	0.44 ± 0.07^a^	1.13 ± 0.05^c^
DHA C22:6 n3	1.27 ± 0.07^a^	12.30 ± 0.05^b^	12.95 ± 0.15^c^
EPA + DPA + DHA	8.40 ± 0.67^a^	31.01 ± 3.88^c^	28.70 ± 2.71^b^
Σ n-3 PUFA	12.12 ± 0.75^a^	31.96 ± 3.90^b^	30.06 ± 2.71^b^
Σ n-6 PUFA	11.50 ± 1.74^b^	8.55 ± 0.47^a^	17.18 ± 3.97^c^
Σ PUFA	23.61 ± 2.50^a^	40.50 ± 4.37^b^	47.27 ± 6.68^c^
Σ n-3 PUFA/Σ n-6 PUFA	1.06 ± 0.10^a^	3.74 ± 0.25^b^	1.76 ± 0.25^c^

Numerical values marked with the same letter ^a, b^ or ^c^ are not statistically significantly different.

*SFA* saturated fatty acids, *MUFA* monounsaturated fatty acids, *PUFA* polyunsaturated fatty acids, *ND* not detected.

### Comparison of vitamin composition

The vitamin composition of isopod meal is relatively comprehensive (Table 5). Among the four fat-soluble vitamins, the content of VA is lower in the isopod than in fish meal, while the contents of VK_1_ and VE (F = 1.81 × 10^5^, df = 2) are much higher than those in krill and fish meal. In addition, the content of water-soluble vitamin VB_2_ (F = 1.30 × 10^5^),VB_3_ (F = 19.13) and VB_5_ is the highest in isopod meal.

**Table 5:** Vitamin composition of isopod meal, Antarctic krill meal and fishmeal.

Vitamin (mg/100 g)	Isopod meal	Krill meal	Fish meal
VA (retinol)	< 0.05	< 0.05	0.19 ± 0.12
VD_3_ (cholecalciferol, μg/100 g)	< 2	< 2	< 2
VK_1_ (phylloquinone, μg/100 g)	64.0 ± 7.45	< 1	< 1
VE (tocopherol)	9.32 ± 0.35^c^	2.53 ± 0.07^b^	0.82 ± 0.02^a^
VB_1_ (thiamine)	ND	0.04 ± 0.01	ND
VB_2_ (riboflavin)	1.68 ± 0.00^b^	0.12 ± 0.02^a^	0.12 ± 0.21^a^
VB_3_ (niacin)	2.83 ± 0.70^b^	1.41 ± 1.02^a^	ND
VB_5_ (pantothenic acid)	2.43 ± 1.29	ND	ND
VB_6_ (pyridoxine, mg/kg)	ND	ND	ND
VB_12_ (cobalamin, mg/kg)	ND	0.849 ± 0.45	ND
Folic acid (mg/kg)	ND	ND	ND
VC (ascorbic acid)	< 1	< 1	< 1

*ND* Not Detected. Values with the same letter ^a, b^ or ^c^ indicates that the differences are not statistically significant between mean values at the *p* < 0.05 level.

### Comparison of mineral composition

The mineral composition of *Ligia exotica* is shown in Table 6. Ubiquitous mineral elements such as calcium (F = 3995.94, all df = 2), potassium (F = 590.06) and magnesium (F = 658.60) are most abundant in the isopod. The trace mineral element ferrum (F = 13185.74), chromium (F = 33.59) and selenium (F = 406.02) are the richest in the isopod, while copper content (F = 160.04) is higher in both isopod and krill meal than that in fishmeal.

**Table 6:** Minerals composition of isopod meal, Antarctic krill meal and fishmeal.

Minerals (mg/kg)	Isopod meal	Krill meal	Fish meal
Calcium	90,283 ± 1536.34^c^	21,536.04 ± 3688.32^b^	18,575.06 ± 2630.42^a^
Potassium	5403.4 ± 148.76^c^	2378.68.15 ± 340.11^a^	3352.11 ± 294.32^b^
Sodium	8117.13 ± 315.41^b^	10,592.23 ± 1528.86^c^	4033.63 ± 1039.12^a^
Magnesium	4862.67 ± 100.74^c^	4517.53 ± 508.37^b^	1256.98 ± 255.49^a^
Copper	31.00 ± 4.97^b^	70.76 ± 18.93^c^	4.74 ± 18.93^a^
Ferrum	882.67 ± 18.31^c^	84.27 ± 25.04^b^	22.10 ± 2.81^a^
Zinc	62.95 ± 6.86^b^	52.04 ± 7.08^a^	74.40 ± 10.48^c^
Chromium	2 ± 0.35	None	None
Selenium	2.69 ± 0.30^c^	1.54 ± 0.32^b^	0.4 ± 0.01^a^
Manganese	60.67 ± 6.26^a^	2.6 ± 0.50^c^	25 ± 4.97^b^
Total phosphorus (%)	0.43 ± 0.05^c^	1.42 ± 0.01^a^	1.15 ± 0.19^b^

Numerical values marked with the same letter ^a,b^ or ^c^are not statistically significant different at the *p* < 0.05 level.

### Comprehensive comparison of amino acids, fatty acids, vitamins and minerals

**Figure 5:**
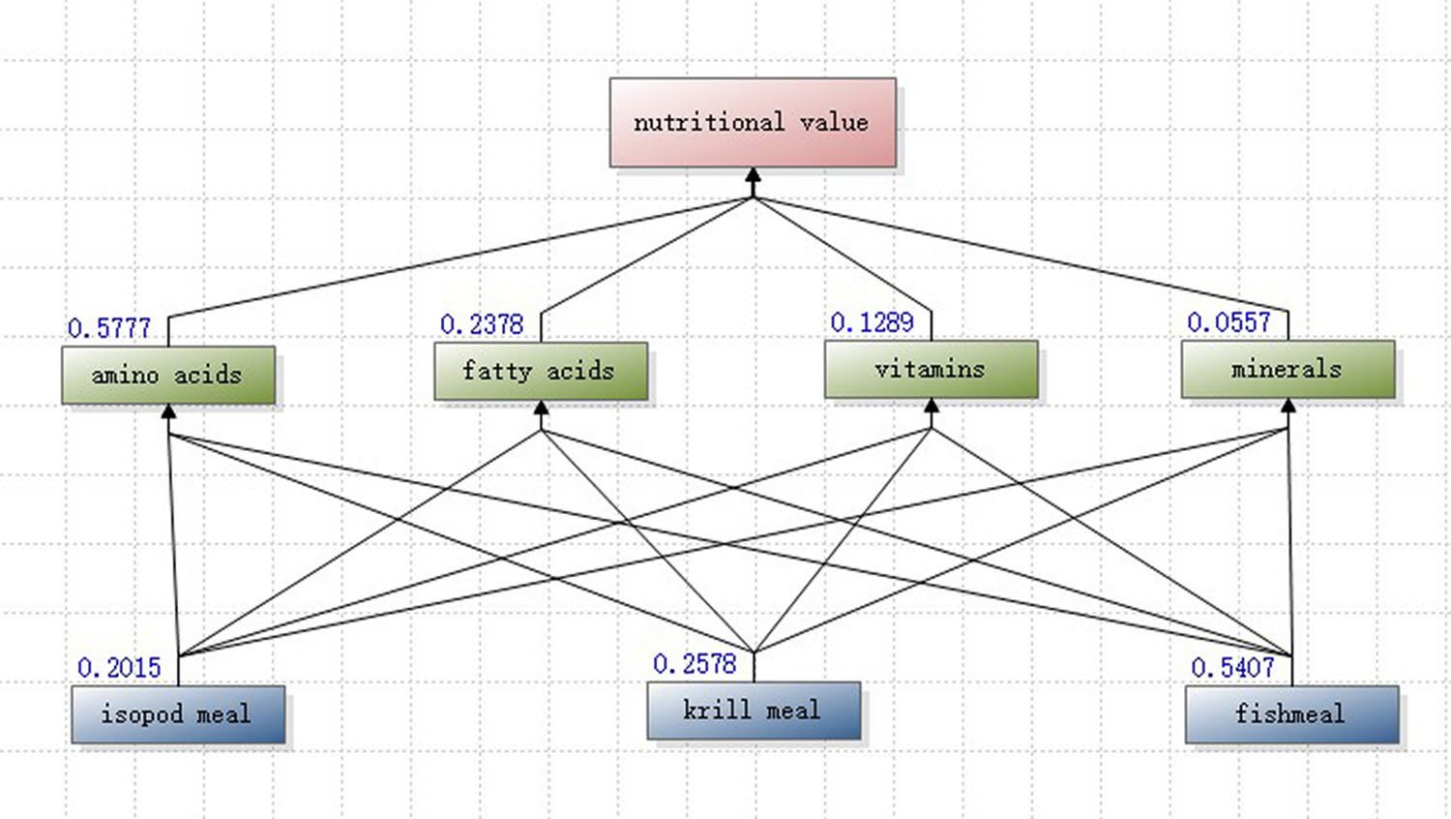
Nutritional value of three substrates based on group decision analytic hierarchy process of experts’ judgement. The larger the blue numbers, the greater the importance of the index. It was computed and generated with software YAAHP (Yet Another Analytical Hierarchy Process, https://www.metadecsn.com/yaahp/). Version 10.0, Shanxi Meta-Decision Software Technology Co. Ltd, China.

Based on the group decision hierarchy process to summarize experts’ judgment, the weighted index nutritional value is shown in blue numbers of Fig. 5.

For human or animal consumption, the importance of amino acids, fatty acids, vitamins and minerals are different. On this basis, fishmeal is the most nutritionally rich substrate (weight = 0.5407), and isopod meal is the least (weight = 0.2015), due largely to the imbalance in nutritional elements.

**Figure 6:**
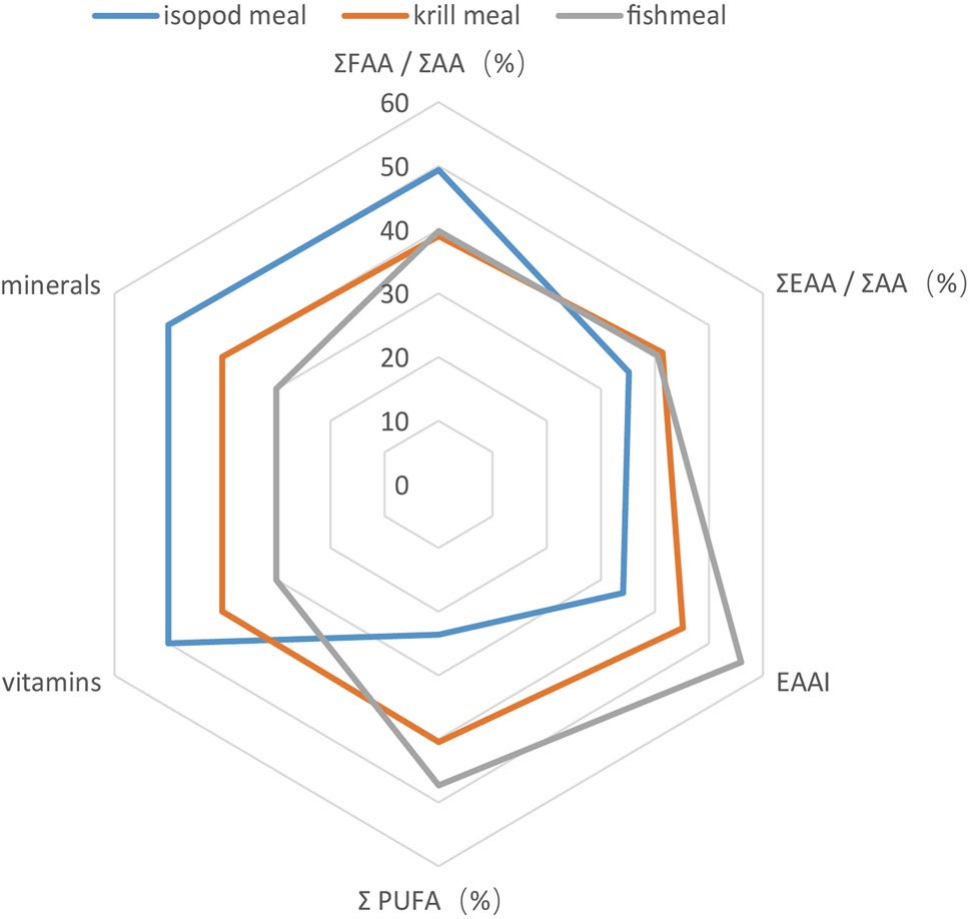
A radar chart illustrating the comprehensive nutritional evaluation of isopod meal, krill meal and fishmeal. Factors of ΣFAA/ΣAA(%),ΣEAA/ΣAA(%), EAAI andΣPUFA were scored based on the actual values from Tables 2, 3 and 4. For other two factors, three food materials are ranked based on the top rank having the highest value in the number of parameters, followed by the second and third respectively. For example, isopod substrate has six highest parameters in the mineral category, followed by krill with two and fishmeal one (Table 6), so they are ranked first, second and third respectively. To facilitate comparison with other factors in the radar chart, numbers "50", "40", "30" were assigned to the first, second and third ranked materials respectively.

In order to better visualize the differences of nutrient composition of the three food materials, a radar chart (Fig. 6) was constructed including essential amino acids, flavor amino acids, essential amino acid index, Σ PUFA, vitamins (eight parameters) and minerals (nine parameters). As can be seen there are clear differences between the assessed nutritional value of isopod, krill and fish meal. Isopod substrate scores better in minerals and vitamin content, and has a certain flavor stimulating effect (based on ΣFAA/ΣAA (%)). However, fatty acid content, especiallyΣPUFA, is far lower than that of krill meal and fish meal. Fish meal scores best in EAAI andΣPUFA. Unsaturated fatty acids (including PUFA) are known to have beneficial physiological functions such as improving blood microcirculation and increasing the activity of brain cells. While the closer the protein composition is to the egg protein, the easier it is absorbed and utilized by humans (view from EAAI). To sum up, the nutritional value of isopod is inferior to that of krill and fish meal.

## Discussion

**Figure 7:**
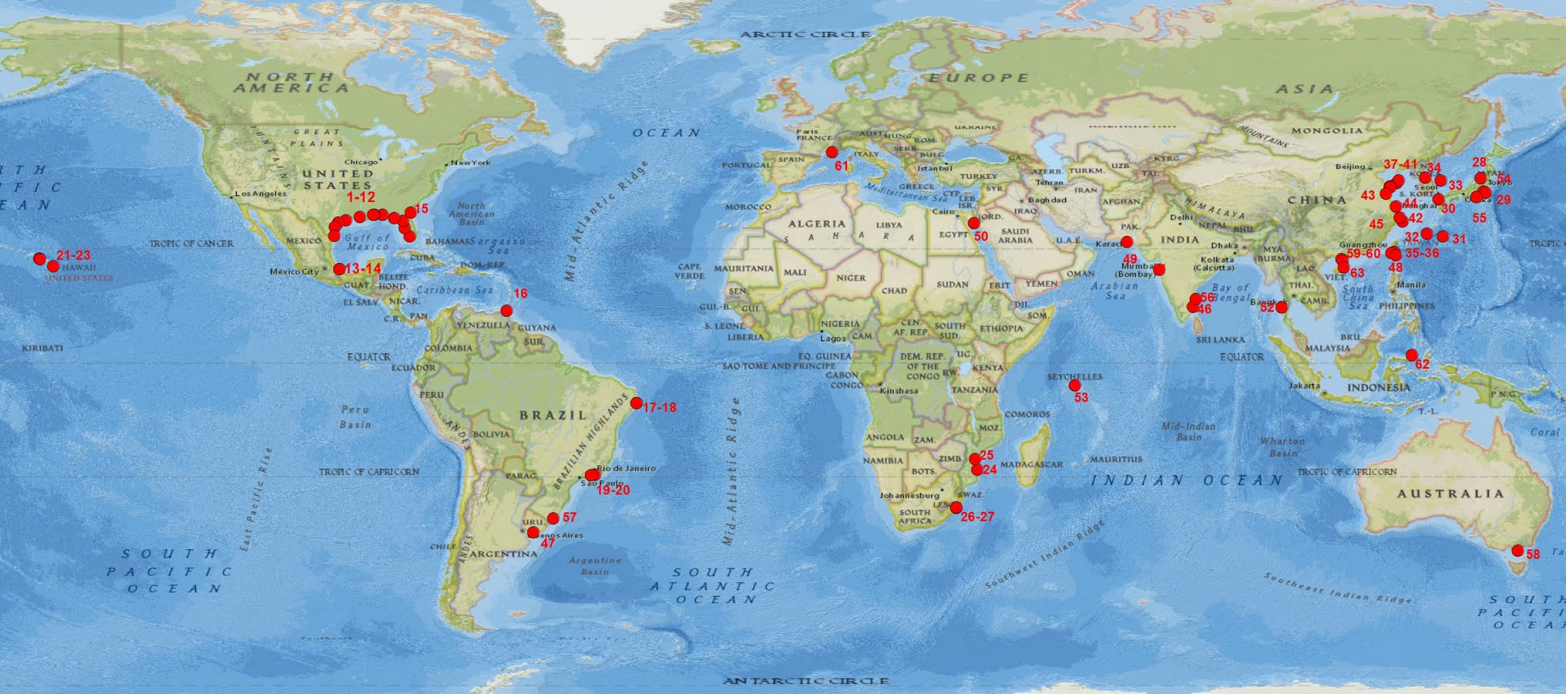
Currently documented global distribution of *L. exotica* (red dots). Mainly adapted from Hurtado et al., 2018. Map source: National Geographic World Map (ESRI). Downloaded from ArcGIS online, 2019. Sample locations were generated with software ArcGIS, desktop standard for business (https://www.esri.com/en-us/arcgis/products/arcgis-desktop/overview). Version 10.5, Esri, USA, from excel based on Supplementary Table S2.

*Ligia* species are distributed globally and *Ligia exotica* is probably the most widely distributed among about 30 species of the genus. Based on the data presented in Hourado et al., 2018^30^, the sites sampled in the current study and after reviewing the literature on *Ligia exotica*, we have compiled a comprehensive list of locations where *Ligia* are known or generally available (see Supplementary Table S2 online) and constructed a global distribution map (Fig. 7) generated by ArcGIS software. This is useful to illustrate the widespread nature of the species and therefore its broad availability as a potential medium for food.

*Ligia exotica* represents one of the oldest documented introductions of marine organisms, originally described by Roux^31^ at the docks in Marseille, France (ID = 61, Fig. 7). This is the northernmost location, while the southernmost location is Sunday Island, Australia^32^ (ID = 58, Fig. 7). They are widely distributed in tropical and temperate regions, including the Seychelles archipelago and Hawaiian Islands suggesting significant colonization ability, but the species have not been found in the Antarctic or Arctic. The southern coast of the United States and the coast of East Asia are two major hot spots. East Asia is traditionally considered to be the origin of *L. exotica*. In China, *L. exotica* is distributed all over the rocky coast^1^, including Taiwan (ID = 35, 36. Figure 7). Areas in eastern China account for 70% of Chinese human population, whose activities not only bring a large amount of nutrients to the coastal waters by way of waste discharge and disposal, but also provide habitats to *L. exotica* in form of wharves and dams. *Ligia* sp. plays an important role as a scavenger/detritivore, feeding on a large range of organic matter plant debris and animal corpses brought by tides.

The only published study on *Ligia* growth we are aware of to date is Carefoot^2^ on the field population and growth of *Ligia pallasii* Brandt. Further work examined the nutritional requirements of *Ligia pallasiii* using artificial diets (consisting of a dry aggregate of 59 chemical substances) and demonstrated that this species is able to grow from 56.5 mg to 111.6 mg over 40 weeks of culture^33^. The resulting specific growth rate (SGR) is only 0.24%. In contrast, for the Pacific white shrimp *Litopenaeus vannamei*, the SGR of genetically selected high growth lines could reach 29.25%^34^. In present study, juvenile *Ligia exotica* has an SGR of 6.97% after 70 days of culture. Juvenile are expected to keep growing for a long time until they mature. Usually isopods live for 1.5 to 2 years. The breeding occurs in the spring and early summer, with some females carrying winter broods of eggs (Carefoot, field observation^3^). Hence it can be deduced the isopods mature sexually around 6 months to 1 year.

In China, crustacean farming for food (represented by species such as Pacific white shrimp *Litopenaeus vannamei* and American crayfish) is a significant industry. The annual output of white shrimp is more than 1.5 million tons, and its gross value is more than 8.7 billion dollars. *Litopenaeus vannamei* also has a mineralised cuticle that sheds regularly to allow for growth. From the aspect of academic research, *Ligia extocia*, given its abundance and large geographic range, has the potential to become a model animal for crustacean studies related to aquaculture, and to better understand some of the physiological properties of crustacea such as the shrimps that are economically important. For example, the calcium translocations and transepithelial movement during the moulting cycle of *L.vannamei*, and dietary calcium requirement in low salinity environments^35^. This undoubtedly has significant theoretical and applied value.

The primary aim of present study was to examine the nutritional value of *Ligia* as a potential new natural food source in aquaculture based on our previous study that confirmed that *L. exotica* provides a good diet for juvenile cuttlefish^21^. In comparison to both krill and fish meal, the nutritional value of protein and amino acids of *Ligia* isopod is lower in almost all evaluation indexes, such as crude protein content, ΣEAA/ΣAA (%), ΣEAA/ΣNEAA (%), and EAAI. In particular the two amino acids with the lowest values for *L. exotica*, methionine and cystine, are present at less than half of that of krill meal and fishmeal. The imbalance of these amino acids may affect the digestion and absorption of predators from isopod food. However, isopods have a relative high value of ΣFAA/ΣAA (%) and the contents of taurine are 4 to 5 times those of fish meal and krill. As a sulfonic acid, taurine is a found in high concentrations in animal tissues and has been attributed a wide diversity of roles for food additives. It is added to cat food, chicken feed, energy drinks, infant formula, dietary supplements, cosmetics, inert ingredients in pesticides and pharmaceuticals^36^. For instance, a number of studies (Salze et al. for review^37^) have demonstrated the essentiality of dietary taurine for many commercially relevant species, especially marine teleosts. In the European Union and China, taurine is authorized for fish feed in all species. Consequently, combining with the flavor amino acid, they may possibly be the most useful ingredients that *L. exotica* provided to develop into food additive, especially in feeding stimulation, for animal culture.

*Ligia* contains more saturated fatty acid (SFA) than fishmeal and krill, but carries fewer polyunsaturated fatty acid (PUFA), which is important food element especially in the cardiovascular health of consumers. This also reduces its potential nutritional value. Interestingly, however, the isopod has superior vitamin content as concentrations of VK_1_, VE, VB_2_, VB_3_ and VB_5_ are all far higher than that in krill and fishmeal. It should be noted that the vitamin content in substrate is highly variable, influenced by several factors, such as origin and composition of the animal, meal processing method, and product freshness^38^. Under the processing methods of this study, the three substrates went through a process of heating and drying at high temperature, so for unstable vitamins such as VC, VB_1_ and folic acid problems with detection may have occurred. In addition, part of fat-soluble vitamins in fish meal were lost during oil extraction.

The mineral composition analyses show that calcium accounts for a very large proportion of body content in the isopod, which is the relatively stable ubiquitous elements. However, the body concentration of metal elements in isopods is highly affected by the intertidal environment, and they have a high tolerance to heavy metal contaminants. In areas with severe anthropogenic contamination, heavy metal elements are concentrated through food chains and can accumulate in isopods^39^. For example, high concentrations of copper in *Ligia* from the Santa Rosalía area are consistent with mining activities at this location^40^. In addition, it has been reported that *Ligia* sp*.* can accumulate harmful organic chemicals, such as POPs^41^, TBT^42^, and even radioactive substances^43^. The sampled area in the present study, Jinsha Bay, Zhanjiang City (ID = 59, Fig. 7) is a hot spot for human activity and waste discharge, therefore it is likely that *Ligia* would be exposed to and accumulate many heavy metal pollutants. Since the intertidal zones are exposed to pollution from both marine and terrestrial sources, isopods could potentially be used as biomonitors of pollution in these habitats in a similar way to terrestrial isopods in soil ecotoxicology^44^. Indeed, Longo et al.^45^ reported that *Ligia italica* play the role of bioindicator for heavy metals pollution in the supralittoral zone. Based on the nutritional analysis reported here, *Ligia* offers the potential as a natural additive for animal food, but *Ligia* should only be sourced from relatively clean environments or from artificial culture.

## Conclusion

To conclude, given the rapid growth rate under culture, acclimatization ability and the fact that it can be cultivated either in or out of water, in addition to the nutritional analysis reported here, it suggests that *Ligia extocia* has potential to serve as an alternative natural food source in aquaculture even animal farming. It is rich in taurine and flavor amino acids and has been confirmed that especially suitable for cuttlefish which prefers to live on crustacean as diet. However, the unbalanced amino acid composition and lower content of PUFA may limit its practical value. Considering its unique semi-terrestrial ability and its role in the material cycle of the coastal zone, further study is warranted to elucidate its biological characteristics as a potential model species and on how *Ligia* diet translates into food quality in animal culture.

## Supplementary Information

Supplementary information.

## Data Availability

The data sets used and analyzed during the current study are available from the corresponding author on reasonable request.

## Amino Acids include

Ile: Isoleucine
Leu: Leucine
Lys: Lysine
Thr: Threonine
Val: Valine
Trp: Tryptophan
Met: Methionine
Cys: Cysteine
Phe: Phenylalanine
Tyr: Tyrosine
AAS: Amino acid score
BW: Body weight
CS: Chemical score
DAH: Days after hatching
EAAI: Essential amino acid index
FAAC: Flame atomic absorption spectrometry
GB: Chinese national determination standard
HPLC: High performance liquid chromatography
ICP-MS: Inductively coupled plasma- mass spectrometry
MUFA: Monounsaturated fatty acids
ND: Not detected
PUFA: Polyunsaturated fatty acids
RP-HPLC: Reversed-phase chromatography
SFA: Saturated fatty acids
SGR: Specific growth rate
ΣAA: Total amino acids
ΣEAA: Total essential amino acids
ΣNEAA: Total nonessential amino acids
