# Supplementary material for: A comprehensive evaluation of the potential of semiterrestrial isopods, Ligia exotica, as a new animal food
Source: Sci Rep. 2021 Mar 30;11:7213. doi: 10.1038/s41598-021-86561-z (PMC8009950; doi:10.1038/s41598-021-86561-z)
Supplement: Supplementary file 1 — Supplementary information. [file 41598_2021_86561_MOESM1_ESM.docx]

**A comprehensive** **evaluation of the** **potential of semiterrestrial isopods, *Ligia exotica*, as a new animal food**

Lele Xu^a,b+^, Yongqin Li^b+^, Yao Liu^c^, Haifeng Mi^d^, Xiang Jiang^a,b^, Yulin Sun^a,b^, Haiyong Zhao^e^, Daohai Chen^a,b^, Liyun Wang^a,b*^

^a^Engineering Technology Research Center for Sustainable Utilization of Seafood Resources in Western Guangdong Province, Zhanjiang, China

^b^Life Science and Technology School, Lingnan Normal University, Zhanjiang, China

^+^ These authors contributed equally to this work

^c^School of Information Engineering, Lingnan Normal University, Lingnan Normal University, Zhanjiang, China

^d^Fisheries Institute, Tongwei Group Co., Ltd. Chengdu, China

^e^School of Foreign Studies, Capital University of Economics and Business, Beijing, China

* E-mail correspondence: wangliyunjp@yahoo.co.jp

Supplementary File

| Nutrient components | Code and name of Chinese national determination standard | | Brief description of methods and equipment |
| --- | --- | --- | --- |
| Moisture | GB 5009.3-2016 | Determination of moisture in feedstuffs | direct drying method |
| Crude protein | GB 5009.5-2016 | Determination of crude protein in feeds | Kjeldahl method |
| Crude ash | GB 5009.4-2016 | Animal feeding stuff- Determination of Crude ash | 550℃in muffle furnace |
| Crude fat | GB 5009.6-2016 | Determination of Crude fat in feeds | Soxhlet extraction |
| Taurine | GB 5009.169.2016 | Determination of taurine in food | neighbor grows responds with o-Phthalaldehyde |
| Amino acids | GB 5009.124-2016 | Determination of amino acids in foods | amino acid analyzer |
| Cystine | GB/T 18246-2000 | Determination of amino acids in feeds | oxidation hydrolysis |
| Tryptophan | GB/T 18246-2000 | Determination of amino acids in feeds | Alkali hydrolysis, RP-HPLC |
| Vitamin A & Vitamin E & Vitamin D_3_ | GB 5009.82-2016 | Determination of vitamin A, D, E in food | RP-HPLC |
| Vitamin B_1_ | GB 5009.84-2016 | Determination of vitamin B_1_ in food | HPLC |
| Vitamin B_2_ | GB 5009.85-2016 | Determination of vitamin B_1_ in food | HPLC |
| Vitamin B_3_ | GB 5009.89-2016 | Determination of niacin in food | HPLC |
| Vitamin B_5_ | GB 5009.210-2016 | Determination of pantothenic acid in food | HPLC |
| Vitamin B_6_ | GB/T 14702-2018 | Determination of vitamin B_6_ in premix | HPLC |
| Vitamin B_12_ | GB/T 17819-2017 | Determination of vitamin B_12_ in additive premix | HPLC |
| Vitamin C | GB 5009.86-2016 | Determination of ascorbic acid in food | HPLC |
| Vitamin K1 | GB 5009.158-2016 | Determination of vitamin K1 in food | HPLC plus fluorescence detection |
| Folic acid | GB/T 17813-2018 | Determination of folic acid in premix | HPLC |
| Nicotinic acid | GB 5009.89-2016 | Determination of niacin and nicotine in food | HPLC |
| Pantothenic acid | GB 5009.210-2016 | Determination of pantothenic acid in food | HPLC |
| Potassium & Sodium | GB 5009.91-2017 | Determination of potassium and sodium in food | FAAS |
| Magnesium | GB 5009.241-2017 | Determination of Magnesium and sodium in food | FAAS |
| Calcium | GB 5009.92-2016 | Determination of calcium in food | FAAS |
| Copper | GB 5009.13-2017 | Determination of copper in food | graphite furnace atomic absorption |
| Chromium | GB 5009.268-2016 | Determination of multiple elements in Food | ICP-MS |
| Ferrum | GB 5009.90-2016 | Determination of multiple elements in Food | Flame atomic absorption spectrometry (FAAS) |
| Zinc | GB 5009.14-2017 | Determination of zinc in food | FAAS |
| Manganese | GB/T 13885-2017 | Determination of the content of manganese in feed | Atomic absorption spectrometry |
| Total phosphorus | GB/T 6437-2018 | Determination of phosphorus in feeds | spectrometry |
| Cholesterol | GB 5009.128-2016 | Determination of cholesterol in food | HPLC |

Supplementary Table S1. Nutritional items to analysis and their determination methods

| **Locality Names** | **ID** | **Sources** | **Lat** | **Long** |
| --- | --- | --- | --- | --- |
| Goodland, FL, USA | 1 | Hurtado *et al*. 2018^1^ | 25°55'57''N | 81°39'21''W |
| Sunshine Skyway Bridge North Rest Area, St. Petersburg, FL, USA | 2 | Hurtado *et al*. 2018^1^ | 27°39'14''N | 82°40'41''W |
| Cedar Key, FL, USA | 3 | Hurtado *et al*. 2018^1^ | 29°8'8''N | 83°2'11''W |
| Eastpoint, FL, USA | 4 | Hurtado *et al*. 2018^1^ | 29°44'21''N | 84°52'25''W |
| Pensacola, FL, USA | 5 | Hurtado *et al*. 2018^1^ | 30°25'11''N | 87°11'36''W |
| Biloxi Small Craft Harbor, Biloxi, MS, USA | 6 | Hurtado *et al*. 2018^1^ | 30°23'31''N | 88°53'8''W |
| Long Beach Harbor, Biloxi, MS, USA | 7 | Hurtado *et al*. 2018^1^ | 30°20'41''N | 89°8'42''W |
| Avery Island, LA, USA | 8 | Hurtado *et al*. 2018^1^ | 29°54'57''N | 91°54'14''W |
| Galveston, TX, USA | 9 | Hurtado *et al*. 2018^1^ | 29°17'43''N | 94°48'28''W |
| Palacios, TX, USA | 10 | Hurtado *et al*. 2018^1^ | 28°44'18''N | 96°24'6''W |
| Municipal Harbor, Port Aransas, TX, USA | 11 | Hurtado *et al*. 2018^1^ | 27°50'24''N | 97°3'50''W |
| South Padre Island, TX, USA | 12 | Hurtado *et al*. 2018^1^ | 26°4'44''N | 97°10'9''W |
| San Juan de Ulúa Fort, Veracruz, Mexico | 13 | Santamaria *et al.* 2013^2^ and Hurtado *et al*. 2018^1^ | 19°12'34''N | 96°7'51''W |
| Jetty by Adolfo Ruiz Cortines statue, Veracruz, Mexico | 14 | Hurtado *et al*. 2018^1^ | 19°11'40''N | 96°7'24''W |
| Cumberland Island, GA, USA | 15 | Wetzer 2001^3^ and Hurtado *et al*. 2018^1^ | 30°51'N | 81°27'W |
| Chaguaramas Bay, Trinidad, Trinidad and Tobago | 16 | Hurtado *et al*. 2018^1^ | 10°40'57''N | 61°37'21''W |
| Praia de Calhetas, Cabo de Santo Agostinho, Brazil | 17 | Hurtado *et al*. 2018^1^ | 8°20'38''S | 34°56'43''W |
| Praia do Paraíso, Pernambuco, Brazil | 18 | Hurtado *et al*. 2018^1^ | 8°21'S | 34°57’W |
| Rio de Janeiro, Brazil | 19 | Hurtado *et al*. 2018^1^ | 23°2'50''S | 43°31'10''W |
| Lagoa Azul, Ilha Grande, Costa Verde, Brazil | 20 | Hurtado *et al*. 2018^1^ | 23°11'S | 44°18'W |
| Hilo Harbor, Hawai’i, HI, USA | 21 | Hurtado *et al*. 2018^1^ | 19°43'57''N | 155°3'26''W |
| Pearl Harbor, O’ahu, HI, USA | 22 | Hurtado *et al*. 2018^1^ | 21°21'50''N | 157°57'37''W |
| Honolulu Harbor, O'ahu, HI, USA | 23 | Taiti *et al*. 2003^4^ and Hurtado *et al*. 2018^1^ | 21°18'9''N | 157°51'53''W |
| Vilankulos, Mozambique | 24 | Hurtado *et al*. 2018^1^ | 21°59'52''S | 35°19'30''E |
| Beira, Mozambique | 25 | Hurtado *et al*. 2018^1^ | 19°50'53''S | 34°53'35''E |
| Durban Harbor, KwaZulu-Natal, South Africa | 26 | Hurtado *et al*. 2018^1^ | 29°52'19''S | 31°1'30''E |
| Blue Lagoon, Umgeni River Mouth, KwaZulu-Natal, South Africa | 27 | Hurtado *et al*. 2018^1^ | 29°48'36''S | 31°2'8''E |
| Niigata, Japan | 28 | Hurtado *et al*. 2018^1^ | 37°54'58''N | 139°2'11''E |
| Kanagawa, Japan | 29 | Hurtado *et al*. 2018^1^ | 35°9'25''N | 139°36'43''E |
| Fukuoka, Japan | 30 | Hurtado *et al*. 2018^1^ | 33°35'N | 130°24'E |
| Kitadaito son, Okinawa, Japan | 31 | Hurtado *et al*. 2018^1^ | 25°56'45''N | 131°17'56''E |
| Okinawa, Japan | 32 | Hurtado *et al*. 2018^1^ | 26°28'46''N | 127°55'40''E |
| Ulleungdo Island, South Korea | 33 | Hurtado *et al*. 2018^1^ | 37°30'6''N | 130°51'11''E |
| Boryeong, South Korea | 34 | Hurtado *et al*. 2018^1^ | 38°4'53''N | 127°38'16''E |
| Lutao, Taitung, Taiwan, China | 35 | Hurtado *et al*. 2018^1^ | 22°45'6''N | 121°9'42''E |
| Pingtung County, Taiwan, China | 36 | Hurtado *et al*. 2018^1^ | 22°29'44''N | 120°36'52''E |
| Rushan, Shandong, China | 37 | Yin *et al*. 2013^5^ | 36°50'59''N | 121°36'50''E |
| Weihai, Shandong, China | 38 | Yin *et al*. 2013^5^ | 37°26'14''N | 122°9'42''E |
| Qingdao-Zhanqiao, Shandong, China | 39 | Yin *et al*. 2013^5^ | 36°3'41''N | 120°19'10''E |
| Qingdao-Hongdao, Shandong, China | 40 | Yin *et al*. 2013^5^ | 36°10'58''N | 120°16'57''E |
| Qingdao, Shandong, China | 41 | Hurtado *et al*. 2018^1^ | 36°3'58''N | 120°22'10''E |
| Zhujiajian Island, Zhejiang, China | 42 | Hurtado *et al*. 2018^1^ | 29°54''N | 122°53'E |
| Lianyungang, Jiangsu, China | 43 | Yin *et al*. 2013^5^ | 34°46'32''N | 119°26'34''E |
| Nantong, Jiangsu, China | 44 | Yin *et al*. 2013^5^ | 32°5'7''N | 121°35'51''E |
| Zhujiazian, Zhoushan Islands, China | 45 | GenBank and Hurtado *et al*. 2018^1^ | 29°52′12″N | 122°23′55″E |
| Parangipetta, India | 46 | Hurtado *et al*. 2018^1^ | 11°29'24''N | 79°45'36''E |
| Punta Carretas, Montevideo, Uruguay | 47 | Hurtado *et al*. 2018^1^ | 34°56'06'' S | 56°09'40'' W |
| Orchid Island, Taiwan | 48 | Hurtado *et al*. 2018^1^ | 22°04'51"N | 121°30'44"E |
| Karachi, Pakistan * | 49 | Kazmi. 1993^6^ | 24°45'6''N | 66°9'42''E |
| Jordan coastline, Gulf of Aqaba, Red Sea * | 50 | Ismail. 1990^7^ | 28°43'8''N | 34°41'28''E |
| Bandra, India * | 51 | Joshi & Bal. 1959^8^ | 19°3'39''N | 72°49'35''E |
| Mergui Archipelago, Myanmar * | 52 | Barnard. 1936^9^ | 11°21'5''N | 98°0'48''E |
| Aldabra, Seychelles Archipelago * | 53 | Ferrara & Taiti, 1985^10^ | 4°40'11''S | 55°28'18''E |
| Pacific seashores Kamogawa, Japan | 54 | Horiguchi *et al*. 2006^11^ | 35°1′ N | 140°1′ E |
| Pacific seashores Shimoda, Japan | 55 | Horiguchi *et al*. 2006^11^ | 34°4′ N | 138°6′ E |
| Adyar Beach, Madras, Tamil Nadu, India * | 56 | Ravindranath. 1974^12^ | 13°0'56''N | 80°16'18''E |
| Patos Lagoon, Rio Grande do Sul, Brazil * | 57 | Lopes *et al*. 2006^13^ and Hurtado *et al*. 2018^1^ | 32°2'44''S | 52°4'39''W |
| Sunday Island, Victoria, Australia * | 58 | Green. 1962^14^ and Hurtado *et al*. 2018^1^ | 38°43'22''S | 146°37'36''E |
| Jinsha Bay, Tiaoshun island, Zhanjiang, China | 59 | This study | 21°16'59''N | 110°24'8''E |
| Naozhou island, Zhanjiang, China | 60 | This study | 20°54'28"N | 110°33'38''E |
| Old Port, Marseille, France * | 61 | Roux P. 1828^15^ and Hurtado *et al*. 2018^1^ | 43°17'38.75''N | 5°21'47.61''E |
| Manado Post, Manado, Indonesia | 62 | Undap *et al*. 2013^16^ | 1°28'22.84''N | 124°49'50.23''E |
| Qinglan port, Hainan, China | 63 | This study | 19°34'1.73''N | 110°49'27.35''E |

Supplementary Table S2. Information on the distribution of *Ligia exotica* worldwide.

(mainly adapted from Hurtado et al., 2018). Note: * means presumed longitude and latitude

**References for Supplementary Table S2**

1. Hurtado, L.A., Mateos, M., & Wang, C., et al. Out of Asia: mitochondrial evolutionary history of the globally introduced supralittoral isopod *Ligia exotica*. *Peer. J.* **6**, 4337 (2018).
2. Santamaria, C.A., Mateos, M., & Taiti, S., et al. A complex evolutionary history in a remote archipelago: phylogeography and morphometrics of the Hawaiian endemic *Ligia* isopods. *Plos One*. **8**, e85199 (2013). doi: 10.1371/journal.pone.0085199.
3. Wetzer, R. Hierarchical analysis of mtDNA variation and the use of mtDNA for isopod systematics (Crustacea: Isopoda). *Contrib. to. Zool*. **70**, 23-39 (2001). https://doi.org/10.1163/18759866-07001002
4. Taiti, S., Arnedo, M.A., Lew, S.E, & Roderick, G.K. Evolution of terrestriality in Hawaiian species of the genus *Ligia* (Isopoda, Oniscidea). *Crustaceana Monographs*, **V**: 85-102 (2003).
5. Yin, J.W. Species assignment and comparative phylogeographic studies on the genus *Ligia* (Crustacea: Malacostraca: Isopoda: Ligiidea) coastally distributed in Chinese Mainland. Nanjing Normal University. Ph D dissertation, China. (2013).
6. Kazmi, Q.B., An illustrated key to the Malacostraca (Crustacea) of the Northern Arabian Sea Part -V: Isopoda. *Pak. J. Mar. Sci*. **2**, 49-66 (1993).
7. Ismail, N.S. Seasonal variation in community structure of macrobentic invertebrates in sandy beaches of Jordan coastline, Gulf of Aqaba, Red Sea. *Int. Rev. Hydrobiol*. **75**, 605–617 (1990).
8. Joshi, U.N., & Bal, D.V. Some of the littoral species of Bombay isopods, with detailed description of two new species. *J. Univ. Bombay*. **27**, 57–69 (1959).
9. Barnard, K.H. Isopods collected by the R.I.M.S. “Investigator”. *Rec. Indian. Mus. Calcutta*. **38**,147–191 (1936).
10. Ferrara, F., & Taiti, S. The terrestrial isopods (Crustacea) of Aldabra. *Zool. J. Linn. Soc*. **86**, 291-215 (1985).
11. Horiguchi, H., Hariyama, T., Takano, S., & Yamagishi, H. Photosensitive neurogenic heart of the isopod crustacean *Ligia exotica*. *Proc. Biol. Sci.* **273**, 2535–2540 (2006).
12. Ravindranath, M.H. The hemocytes of an isopod *Ligia exotica* Roux. *J. Morphol*. **144**, 11-21 (1974). https://doi.org/10.1002/jmor.1051440103
13. Lopes, E.R.C., Blasina, J.R., Dumont, L.F.C., & D'Incao, F. Reproductive biology of *Ligia exotica* (Crustacea, Isopoda, Ligiidae) in Rio Grande, Rio Grande do Sul State, Brazil. *Iheringia Serie Zoologia*. **96**, 5-12 (2006).
14. Green, A.J.A. Record of the occurrence in Australia of *Ligia exotica* Roux (Crustacea, Isopoda, Oniscoidea). *Papers and Proceedings of the Royal Society of Tasmania*. **96**, 83-85 (1962).
15. Roux, P. Crustacés de la Méditerranée et de son littoral. Marseille: Levrault. (1828).
16. Undap, S. L. Matsunaga, S. Hond, M. et al. Accumulation of organotins in wharf roach (*Ligia exotica* Roux) and its ability to serve as a biomonitoring species for coastal pollution. *Ecotoxicol. Environ. Saf*. **96**, 75-79 (2013). https://doi.org/10.1016/j.ecoenv.2013.06.019.
